# Total cholesterol/HDL-C ratio versus non-HDL-C as predictors for ischemic heart disease: a 17-year follow-up study of women in southern Sweden

**DOI:** 10.1186/s12872-021-01971-1

**Published:** 2021-04-05

**Authors:** Susanna Calling, Sven-Erik Johansson, Moa Wolff, Jan Sundquist, Kristina Sundquist

**Affiliations:** Center for Primary Health Care Research, Clinical Research Centre, Department of Clinical Sciences in Malmö, Lund University, Box 50332, 202 13 Malmö, Region Skåne Sweden

**Keywords:** Hyperlipidemias, Lipoproteins, Cholesterol, Non-HDL cholesterol, Ischemic heart disease, Women

## Abstract

**Background:**

A distorted blood lipid profile is an important risk factor for ischemic heart disease (IHD) but the predictive ability of the different lipid measures has rarely been studied. Our aim was to examine and compare, in a large sample of women, the predictive ability of total cholesterol/HDL cholesterol ratio (TC/HDL-C) and non-HDL-C in relation to IHD, adjusted for age, exercise, smoking, waist-hip ratio, blood pressure, and diabetes mellitus.

**Methods:**

Between 1995 and 2000, a total of 6537 women aged 50–59 years from the Women’s Health in Lund area (WHILA) study in southern Sweden were included and underwent a baseline examination. The women were followed through national registers for incidence of IHD during a mean follow-up of 17 years. The prediction accuracy was estimated through Harrell’s C and Akaike Information Criterion (AIC).

**Results:**

Increasing TC/HDL-C as well as non-HDL-C showed strong associations with IHD, with the highest risk in the 5th quintile, where the HR was 2.30 (95% CI: 1.70–3.11) for TC/HDL-C and 1.67 (95% CI: 1.25–2.24) for non-HDL-C, after adjustments. Comparisons using Harrell’s C and AIC indicated that TC/HDL-C has a slightly higher predictive ability than that of non-HDL-C (Harrell’s C 0.62 and 0.59 respectively, *p* = 0.003 for difference, age-adjusted model; AIC for TC/HDL-C < AIC for non-HDL-C).

**Conclusions:**

TC/HDL-C ratio and non-HDL-C are both clinical predictors for IHD in middle-aged women. The results indicate that the predictive ability of TC/HDL-C was higher than that of non-HDL-C; however, non-HDL-C was linearly related to IHD (*p* = 0.58) and may be easier to calculate and interpret in clinical practice, for early identification of future IHD in women.

## Background

The incidence of ischemic heart disease (IHD) among women has increased in several Western countries [1, 2]. Compared with men, the diagnosis of IHD occurs later in life and the symptoms are more ambiguous, which results in underdiagnosis and undertreatment [3, 4]. More knowledge of predictive and modifiable risk factors in middle-aged women is therefore needed in order to identify high-risk individuals and introduce preventive actions at an earlier stage [4, 5].

Blood cholesterol is regarded as one of the most important risk factors for IHD, but the recommendations for clinical use of different lipid measures to predict cardiovascular risk have changed over the years and still diverge [6, 7]. Risk assessment models are useful for clinicians, especially in primary care, to identify high-risk individuals, but the models differ in the choice of lipid measure [8]. Total cholesterol is widely used in Sweden and many other countries [9], but European and American guidelines also recommend non-high-density-lipoprotein-cholesterol (non-HDL-C) for cardiovascular risk assessment, especially in people with diabetes mellitus, obesity or low levels of low-density-lipoprotein-cholesterol (LDL-C) [10–12]. As non-HDL-C includes both LDL-C, very low-density lipoprotein cholesterol (VLDL-C) and intermediate lipoproteins, non-HDL-C is likely to be more atherogenic than either lipoprotein alone and may therefore have a higher predictive ability of IHD [10, 13]. A recent multinational study found that increasing concentrations of non-HDL-C can predict long-term cardiovascular risk, particularly when the increase is modest at a young age [14]. Other studies have suggested that the ratio of total cholesterol (TC) and HDL-C (TC/HDL-C) is the most efficient IHD predictor [15–17]. In a previous study of the same cohort, we showed that TC/HDL-C was a strong predictor for acute myocardial infarction in middle-aged women [18]. However, the clinical use of TC/HDL-C ratio is not widely spread and long-term studies of non-HDL-C and TC/HDL-C in relation to IHD in women are relatively scarce [6]. A more comprehensive picture of cholesterol measures is therefore needed for prediction of IHD risk in middle-aged women.

The first aim was to examine the association between both TC/HDL-C and non-HDL-C and IHD during a 17-year long follow-up of a large sample of middle-aged women, adjusting for a comprehensive set of potential confounders, i.e. age, exercise, smoking, waist-hip ratio, diabetes mellitus type 2 and blood pressure. The second aim was to compare the predictive ability for IHD between TC/HDL-C and non-HDL-C. We also estimated the 5-, 10- and 15-year risks of IHD after baseline adjustments.

## Methods

Data for this cohort study is based on the Women’s Health in Lund Area (WHILA) study, which is a prospective cohort study of middle-aged women in southern Sweden. The details of the study have been described previously [3, 19]. Briefly, between 1995 and 2000, a total of 6916 women aged 50–59 years underwent a health survey after prior invitation to the study. Due to missing values in several variables and exclusion of those with IHD before baseline, a sample of 6537 women was included in the present study. After written consent, a physical examination with measurement of minimal waist and maximal hip circumference was performed. Blood pressure (mm Hg) was measured twice in the right arm, after 15 min and 20 min rest in sitting position, and the average was used. Non-fasting serum levels of TC and HDL-C were measured with a Cholestech LDX-instrument (Cholestech Corporation, Hayward, CA, USA) on capillary whole blood [19, 20]. Furthermore, the women completed a self-administered questionnaire about medical history, medication, lifestyle and sociodemographic data. The study was approved by the Regional Ethical Review Board in Lund (approval no. 2011/494).

### Follow-up and outcome variable

The women were followed from the day of screening until first hospitalization for IHD, through linkage of the data to the Hospital Discharge Register, or until the end of the study on May 31^st^, 2015. The mean and the median follow-up time were 16.3 years and 17.2 years, respectively.

*Ischemic heart disease (IHD)* was based on a diagnosis documented in the Hospital Discharge Register according to the International Classification of Diseases (ICD), i.e.: I20–I25 (ICD-10) or 410–414 (ICD-8/9). We excluded all women who reported a previous diagnosis of IHD at screening (n = 111).

### Predictor variables

*Ratio of total cholesterol (mmol/L) to HDL-C (mmol/L), TC/HDL-C* was categorised into five levels: 1st quintile, 2nd quintile, 3rd quintile, 4th quintile and 5th quintile.

*Non-HDL-C*, calculated as *total cholesterol (mmol/L)* minus *HDL-C (mmol/L), was also* categorised into quintiles.

### Explanatory variables

The explanatory variables were based on the examinations and questionnaires at baseline.

*Agec,* age at screening, continuous, centred around its mean (56 years).

*Exercise* was categorised into Low (only short walks or garden work), Medium (2–4 h walk per week, light level) and High ≥ 2 h per week (strenuous level).

*Waist hip ratio (WHR)* was calculated as waist circumference (cm) divided by hip circumference (cm) and categorised into two groups, ≤ 0.78 and > 0.78 [21]. The cut-off point was chosen based on the distribution in the study sample [18].

*Blood pressure* was categorised into three levels, based on the distribution: (1) systolic blood pressure < 140 mmHg and diastolic blood pressure < 90 mmHg, (2) systolic blood pressure 140–149 mmHg or diastolic blood pressure 90–99 mmHg, and (3) systolic blood pressure ≥ 150 mmHg or diastolic blood pressure ≥ 100 mmHg. The reason for using this categorization instead of using the clinical guidelines for grades of hypertension was because of the distribution and the sample size, as described previously [3, 18].

*Smoking* was categorised into (1) non-smoker (2) former smoker and (3) daily smoker.

*Self-reported Diabetes mellitus type 2* (DM type 2) was based on the questionnaire and dichotomised into Yes/No.

### Statistical method

The response rate varied in the different age-groups between 58.9% (youngest) and 66.7% (oldest), the average was 64.2%. We used post-stratification weights [22] in order to reduce the sampling error and possible non-response bias. The weights were constructed using information on age-group, and municipality at the population level. Thus, we weighted data by age and municipality, w = Ni/ni for responders (Ni = stratum size on population level and ni = number of responders in each stratum) per one-year age-group (50–59 years) and municipality. The weights sum up to the population size in 1995.

In order to analyse the association between the ratio TC/HDL-C as well as non-HDL-C and IHD, we applied Cox regression models; one model was adjusted for age and one model for all the potential confounders (full model), i.e. agec, exercise, smoking, WHR, blood pressure, and diabetes. As menopausal status may be related to lipid changes, we included this variable (yes/no) as a potential confounder. However, the HRs did not change in any of the full models and menopausal status was therefore not included in the final model. All included variables satisfied the proportional hazard assumption. There were no interactions between the ratio of TC and HDL-C (non-HDL-C) and any of the other included covariates. The continuous relationship of TC/HDL-C and non-HLD-C with IHD was estimated by restricted cubic splines, based on full models. As reference levels we chose 4 and 3.5, respectively, both close to their means. The linearity was assessed by applying the command nlcheck in STATA.

We used Harrell’s C and Akaike Information Criterion (AIC) to evaluate the comparison of the predictive ability between TC/HDL-C and non-HDL-C in the different models. Lower values of AIC and higher values of Harrel’s C corresponded to higher risk prediction.

STATA version 16.1 [23] was used for the statistical analyses.

### Analysis of partial non-responders (excluded individuals due to missing values)

We compared the distribution of the different variables by the included individuals (responders) and the excluded individuals that had missing values for one or more variables (partial non-responders) and found significance in smoking (more former smokers among partial non-responders). Responders had a higher risk for IHD than partial non-responders in a Cox regression model adjusted for age. However, the exclusion of partial non-responders should only influence the results to a lesser extent, as they were only approximately 5% of the original sample.

## Results

The baseline characteristics of the women are shown by the categorised variables of TC/HDL-C (Table 1) and non-HDL-C (Table 2). The women were, on average, 56 years of age at the screening and 80% were menopausal. Smoking, high WHR, high blood pressure, diabetes, and low level of exercise were more prevalent in the higher categories of TC/HDL-C and non-HDL-C, respectively. During the mean follow-up period of 17 years, a total of 551 women (8.4%) suffered from IHD. The means of TC and TC/HDL-C were higher in the higher quintiles, and HDL-C was lower (Table 3).

**Table 1:** Distribution (%; mean(sd)) of IHD and the confounders by category of TC/HDL-C at baseline

Variable	TC/HDL-C category					
	Totals	Quintile 1	Quintile 2	Quintile 3	Quintile 4	Quintile 5
**Number of women (%)**	6537	1307 (20.0)	1307 (20.0)	1308(20.0)	1307 (20.0)	1308 (20.0)
**Number of IHD (%)**	551 (8.4)	64(4.9)	91(7.0)	109 (8.3)	104 (8.0)	183(14.0)
**Age, mean (sd)**	56.4 (3.0)	56.0 (2.9)	56.2 (3.0)	56.6 (3.0)	56.6 (3.0)	56.7 (3.1)
**Exercise**						
Low (%)	5.7	3.8	4.7	5.6	5.4	8.9
Medium (%)	51.7	46.0	49.8	50.6	54.8	57.3
High (%)	42.6	50.2	45.5	43.8	39.8	33.8
**Smoking**						
Non-smoker (%)	59.3	63.4	60.7	63.5	57.5	51.2
Former smoker (%)	20.0	21.7	22.0	18.2	19.7	18.6
Daily smoker (%)	20.7	14.9	17.3	20.3	22.8	30.2
**WHR**						
Small (≤ 0.78) (%)	55.0	73.6	64.5	58.6	48.0	30.2
Large (> 0.78) (%)	45.0	26.4	35.5	41.4	52.0	69.8
**Blood pressure**						
SBP < 140 & DBP < 90 mmHg (%)	52.3	59.5	57.6	52.1	49.0	43.3
SBP 140–149 or DBP 90–99 mmHg (%)	27.0	23.3	24.6	28.1	29.0	30.2
SBP ≥ 150 or DBP ≥ 100 mmHg (%)	20.7	17.2	17.8	19.8	22.0	26.5
**Diabetes (self-reported)**						
Yes (%)	2.1	1.2	1.4	2.4	1.9	3.8
No (%)	97.9	98.8	98.6	97.9	98.1	96.2

Distribution (means and number (%)) of IHD and the confounders by category of TC/HDL-C at baseline, n = 6537. TC, total cholesterol; HDL, high-density-lipoprotein cholesterol; TC/HDL-C, total-cholesterol-to-HDL ratio; WHR, waist-hip-ratio; SBP, systolic blood pressure; DBP, diastolic blood pressure

**Table 2:** Distribution (%; mean(sd)) of IHD and the confounders by category of non-HDL-C at baseline

Variable	Non-HDL-C category					
	Totals	Quintile 1	Quintile 2	Quintile 3	Quintile 4	Quintile 5
**Number of women (%)**	6537	1304 (20.0)	1306 (20.0)	1302 (19.9)	1309 (20.0)	1316 (20.1)
**Number (%) of IHD**	551 (8.4)	73(5.6)	92(7.0)	101 (7.8)	133 (10.2)	152 (11.6)
**Age, mean (years)**	56.4 (3.0)	55.9 (2.9)	56.2 (3.0)	56.4 (3.0)	56.6 (3.0)	56.9 (3.1)
**Exercise**						
Low (%)	5.7	4.3	5.4	5.5	6.3	6.8
Medium (%)	51.7	48.5	51.4	51.2	53.4	54.0
High (%)	42.6	47.2	43.2	43.2	40.3	39.2
**Smoking**						
Non-smoker (%)	59.3	62.9	59.4	61.7	58.2	54.2
Former smoker (%)	20.0	21.8	19.8	20.0	20.6	18.1
Daily smoker (%)	20.7	15.3	20.8	18.3	21.2	27.7
**WHR**						
Small (≤ 0.78) (%)	55.0	67.9	63.6	54.2	49.1	40.2
Large (> 0.78) (%)	45.0	32.1	36.4	45.8	50.9	59.8
**Blood pressure**						
SBP < 140 & DBP < 90 mmHg (%)	52.3	61.1	57.5	51.6	48.6	42.6
SBP 140–149 or DBP 90–99 mmHg (%)	27.0	22.9	23.7	27.7	29.3	31.5
SBP ≥ 150 or DBP ≥ 100 mmHg (%)	20.7	16.0	18.8	20.7	22.1	25.9
**Diabetes (self-reported)**						
Yes (%)	2.1	1.4	2.1	1.6	2.8	2.7
No (%)	97.9	98.6	97.9	98.4	97.2	97.3

Distribution (means and number (%)) of IHD and the confounders by category of non-HDL-C at baseline, n = 6537. TC, total cholesterol; HDL, high-density-lipoprotein cholesterol; non-HDL-C, total-cholesterol minus HDL-C; WHR, waist-hip-ratio; SBP, systolic blood pressure; DBP, diastolic blood pressure

**Table 3:** Means (sd) of TC, HDL-C, TC/HDL-C and non-HDL-C by quintiles of TC/HDL-C and non-HDL-C

Variable	Totals	Quintile 1	Quintile 2	Quintile 3	Quintile 4	Quintile 5
**TC/HDL-C category**						
Number of women (%)	6537	1307 (20.0)	1307 (20.0)	1308(20.0)	1307 (20.0)	1308 (20.0)
Ratio TC/HDL-C (mean)	3.7 (1.3)	2.4 (0.3)	2.9 (0.1)	3.4 (0.1)	4.0 (0.2)	5.6 (1.3)
TC (mmol/L)	6.0 (1.1)	5.2 (0.9)	5.7 (0.9)	6.0 (0.9)	6.2 (1.0)	6.7 (1.1)
HDL-C (mmol/L)	1.7 (0.4)	2.2 (0.3)	1.9 (0.3)	1.8 (0.3)	1.5 (0.3)	1.2 (0.2)
**Non-HDL-C category**						
*Number of women (%)*	*6537*	*1304 (20.0)*	*1306 (20.0)*	*1302 (19.9)*	*1309 (20.0)*	*1316 (20.1)*
*Non-HDL-C (mmol/L)*	*4.2 (1.1)*	*2.8 (0.5)*	*3.6 (0.2)*	*4.1 (0.2)*	*4.7 (0.2)*	*5.9 (0.7)*
*TC (mmol/L)*	*6.0 (1.1)*	*4.7 (0.6)*	*5.4 (0.4)*	*5.8 (0.4)*	*6.4 (0.4)*	*7.4 (0.8)*
*HDL-C (mmol/L)*	*1.7 (0.4)*	*1.9 (0.4)*	*1.8 (0.4)*	*1.7 (0.4)*	*1.7 (0.4)*	*1.6 (0.4)*

Means (sd) for TC, HDL-C, TC/HDL-C and non-HDL-C by quintiles of TC/HDL-C category and *non-HDL-C category (in italics)*. N = 6537 and IHD = 551

TC, total cholesterol, HDL-C, high-density-lipoprotein cholesterol, TC/HDL-C, total-cholesterol-to-HDL-C ratio, non-HDL-C, total-cholesterol minus HDL-C

*p* < 0.05 was considered statistically significant

In Table 4, hazard ratios (HR) of TC/HDL-C and non-HDL-C for IHD are presented, with adjustments for age and for all the included variables. We found an increasing association by quintile between TC/HDL-C ratio and IHD and between non-HDL-C and IHD. The highest HR in quintile 5 was 2.30 (95% CI: 1.70–3.11) for TC/HDL-C and 1.67 (95% CI: 1.25–2.24) for non-HDL-C, after adjustments for all the included variables.

**Table 4:** Hazard ratios with 95% confidence intervals of IHD, in age-adjusted models and full models

IHD (age-adjusted models)			IHD (full models*)		
Variable	HR	95% CI	Variable	HR	95% CI
Age centered (age-56)	1.07	1.04–1.10	Age centered (age-56)	1.07	1.04–1.10
TC/HDL-C category			TC/HDL-C category		
Quintile 1	1	Reference	Quintile 1	1	Reference
Quintile 2	1.42	(1.03–1.95)	Quintile 2	1.36	(0.98 -1.88)
Quintile 3	1.65	(1.21–2.25)	Quintile 3	1.54	(1.13–2.11)
Quintile 4	1.56	(1.15–2.14)	Quintile 4	1.38	(1.00–1.89)
Quintile 5	2.92	(2.19–3.89)	Quintile 5	2.30	(1.70–3.11)
*Non-HDL-C category*			*Non-HDL-C category*		
*Quintile 1*	*1*	*Reference*	*Quintile 1*	*1*	*Reference*
*Quintile 2*	*1.22*	*(0.90–1.67)*	*Quintile 2*	*1.15*	*(0.84–1.57)*
*Quintile 3*	*1.34*	*(0.99–1.81)*	*Quintile 3*	*1.24*	*(0.91–1.68)*
*Quintile 4*	*1.72*	*(1.29–2.30)*	*Quintile 4*	*1.50*	*(1.12–2.02)*
*Quintile 5*	*2.03*	*(1.53–2.69)*	*Quintile 5*	*1.67*	*(1.25–2.24)*

HRs with 95% CIs of IHD, in age-adjusted models and full models in relation to categories of TC/HDL-C and *non-HDL-C (in italics)*, n = 6537; IHD = 551

*IHD* ischemic heart disease, *TC/HDL-C* total cholesterol/high density lipoprotein cholesterol, *non-HDL-C* total-cholesterol minus HDL-C, *HR* hazard ratio, *CI* confidence interval

*Adjusted for all included variables: age, also waist hip ratio, smoking, exercise, diabetes mellitus type 2 and blood pressure. *p* < 0.05 was considered statistically significant

**Fig. 1:**
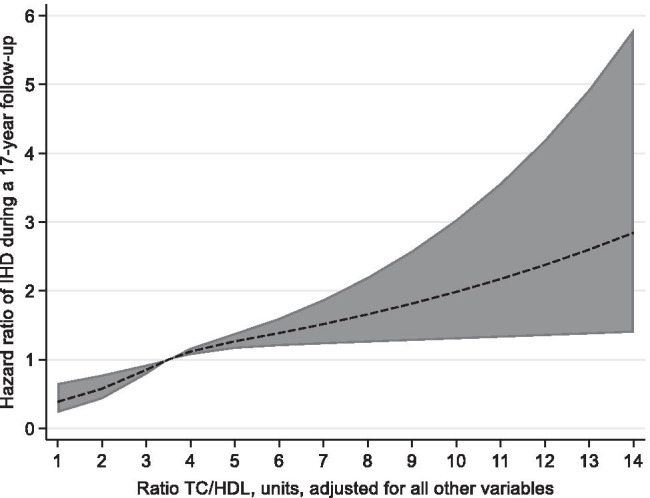
Hazard ratios (HR) with 95% confidence interval (CI) for IHD and TC/HDL_C ratio. Legend: HR and CI estimated by restricted cubic splines. The model is adjusted for all other variables. Reference level HR = 1: Ratio TC/HDL_C = 3.5

**Fig. 2:**
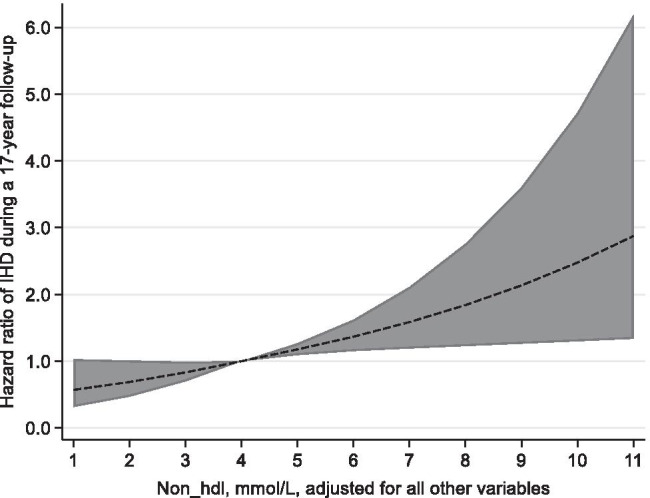
Hazard ratios (HR) with 95% confidence interval (CI) for IHD and non_HDL. Legend: HR and CI estimated by restricted cubic splines. The model is adjusted for all other variables. Reference level HR = 1: non-HDL = 4

The continuous relationships of TC/HDL-C and non-HDL-C with IHD are shown in Fig. 1 and Fig. 2. We found that non-HDL-C was linearly related to IHD (*p* = 0.58); however, TC/HDL-C was not to the same extent linearly related to IHD (*p* = 0.07).

In Table 5 the models are evaluated by Harrell’s C, to assess the IHD predictive ability of the lipid measures. There was a significant difference between TC/HDL-C and non-HDL-C in the age-adjusted models (*p* = 0.003), which indicates that TC/HDL-C has a higher predictive ability for IHD than non-HDL-C, but this difference disappeared in the full model (*p* = 0.061). Moreover, the full models had a significantly higher predictive ability than the age-adjusted models. The AIC test also showed a better fit (higher predictive ability) for TC/HDL-C than for non-HDL-C in the age-adjusted models (AIC was smaller for TC/HDL-C than for non-HDL-C).

**Table 5:** Comparison of IHD predictivity between TC/HDL-C and non-HDL-C shown as Harrell’s C, ΔC and AIC

Test	Age-adjusted model TC/HDL-C	Full model* TC/HDL-C	Δ C (p-value) Full model vs age-adjusted model
n	6537	6537	
Harrell’s C	0.62	0.65	0.028 (*p* < 0.001)
AIC	9472	9431	

Test	Age-adjusted non-HDL-C	Full model* non-HDL-C	Δ C (p-value) Full model vs age-adjusted model
*Harrell’s C*	*0.59*	*0.64*	*0.042 (p < 0.001)*
*AIC*	*9508*	*9453*	
Comparisons of predictivity			
Δ C (p-value) TC/HDL-C vs. non-HDL-C	0.024 (*p* = 0.003)	0.01 (*p* = 0.061)	
AIC comparison	AIC TC/HDL-C < AIC non-HDL-C	AIC TC/HDL-C < AIC non-HDL-C	

*IHD* ischemic heart disease, *TC/HDL-C* total cholesterol/high density lipoprotein cholesterol, *non-HDL-C* total-cholesterol minus HDL-C, *AIC* Akaike Information Criterion

*Adjusted for all included variables: agec, waist hip ratio, smoking, exercise, diabetes mellitus type 2 and blood pressure. *p*<0.05 was considered statistically significant

In Table 6, the risk of IHD (%) at 5, 10 and 15 years is presented by quintile of TC/HDL-C and non-HDL-C. The risk calculation is based on the full models and adjusted for baseline values, i.e. it shows the independent effect of the cholesterol measures. The risks at 5, 10 and 15 years increased by increasing level of TC/HDL-C and non-HDL. The risk of IHD at 15 years was 7.0%; *9.0%* in quintile 4 (TC/HDL-C; *non-HDL-C*) and 12.9%; *10.6%* in quintile 5.

**Table 6:** Risk of IHD (%) by TC/HDL-C and non-HDL-C at 5, 10 and 15 years, respectively

	Risk at 5 years (%)	Risk at 10 years (%)	Risk at 15 years (%)
Full models by Table 4			
**TC/HDL-C**			
Quintile 1	0.9	2.4	4.4
Quintile 2	1.3	3.4	6.2
Quintile 3	1.6	4.1	7.5
Quintile 4	1.5	3.9	7.0
Quintile 5	2.9	7.2	12.9
***Non-HDL-C***			
*Quintile 1*	*1.1*	*2.8*	*5.1*
*Quintile 2*	*1.4*	*3.5*	*6.3*
*Quintile 3*	*1.5*	*3.8*	*7.0*
*Quintile 4*	*2.0*	*5.0*	*9.0*
*Quintile 5*	*2.3*	*5.9*	*10.6*

*IHD* ischemic heart disease, *TC/HDL-C* total cholesterol/high density lipoprotein cholesterol, *non-HDL-C* total-cholesterol minus HDL-C

Risks based on full models, adjusted for baseline age, waist hip ratio, smoking, exercise, diabetes mellitus type 2 and blood pressure

*p* < 0.05 was considered statistically significant

## Discussion

In this cohort study of middle-aged women with a mean follow-up of 17 years, we found a strong association with IHD for both TC/HDL-C ratio and non-HDL-C, after adjustment for age, exercise, smoking, waist-hip ratio, blood pressure and diabetes. The results showed that the higher TC/HDL-C or non-HDL-C, the higher the risk of IHD. Moreover, in comparisons between the two lipoprotein measures, we found that TC/HDL-C had a slightly higher predictive ability for IHD than non-HDL-C. However, this difference disappeared when other confounding factors were taken into account. The absolute risk of IHD over the 15 years of follow-up was 12.9% and 10.6%, respectively, in quintile 5. The strong association indicates that these lipoprotein measures are an important part of cardiovascular risk assessment and a cornerstone of preventive efforts to middle-aged women in clinical practice.

Previous studies have mostly had a shorter follow-up or studied older men and women individuals than in the present study [6, 14]. A cohort study of American middle-aged women, who were followed for 10 years, found that non-HDL-C and TC/HDL-C had higher predictive ability of cardiovascular events than total cholesterol [24], which is recommended by SCORE guidelines [25]. In contrast, a study of older adults (mean age 69 years) found that non-HDL-C was not associated with IHD in women, while TC/HDL-C was [26]. Our results are in line with a multinational study that was published in Lancet in 2019, which analysed the long-term relationship between non-HDL-C and cardiovascular disease in middle-aged individuals, and found that the cardiovascular risk was strongly differentiated by non-HDL-C levels, particularly beyond 10 years [14]. By use of a derivation and validation design, the study simulated a primary preventive risk-modelling, and found that a 50% reduction of non-HDL-C was associated with reduced risk of cardiovascular disease by the age of 75 years, and the earlier the cholesterol was reduced, the greater was the risk reduction. Another meta-analysis also found an age-dependent association between IHD and non-HDL-C and IHD and TC/HDL-C, where the risk increase was higher in younger individuals [27].

Some previous studies have stated that TC/HDL-C is the best predictor for IHD [27, 28] and others have preferred non-HDL-C [14]. A comparison between the two measures has only been addressed in a few previous studies. One UK study made a similar comparison as we did with AIC, and found that TC/HDL-C is a stronger predictor of CHD risk than non-HDL-C, but this study was restricted to men and women with type 2 diabetes [29]. Another study of men and women from the multicenter Atherosclerosis Risk in Communities (ARIC) study found that 21% of individuals with low non-HLD-C had high levels of TC/HDL-C, which was associated with a 29% greater risk of incident atherosclerotic cardiovascular disease [30]. The results of the present study indicate that the predictive ability of TC/HDL-C is higher than that of non-HDL-C for IHD in women, but the difference was not large, which means that either of the lipid measures may be used in clinical practice.

We believe that the results of the present study have relevant clinical implications. Some women have high total cholesterol and LDL-C, but also high HDL-C. For these women, it is important to calculate TC/HDL-C or non-HDL-C to predict their cardiovascular risk more accurately, as the risk may not be increased at all. These women probably do not benefit from, e.g. medication with statins. The recommendations for which lipid measure to use for cardiovascular risk prediction has changed over the years, and in many countries total cholesterol is widely used, e.g. in the SCORE risk prediction model [25]. Early identification of high-risk individuals is important to be able to offer preventive efforts, and long-term risk prediction models are of importance [31]. A recent systematic review of cardiovascular disease risk prediction models identified 363 different models, and total cholesterol was the most common measure for blood lipids, followed by HDL-C [8]. The more recently developed risk prediction model from the PREDICT study in New Zealand, uses TC/HDL-C ratio instead of total cholesterol, and the study concluded that older risk prediction models need to be updated to better fit the modern population [17].

The role of lipoproteins in the development of IHD involves lipid deposition in the arterial wall and the growth and progression of atherosclerotic plaques, with subsequent plaque rupture and formation of an arterial thrombosis [11]. The cumulative exposure to lipoproteins is probably related to the total atherosclerotic plaque burden (concentration and duration); thus, a healthy lifestyle and, sometimes, lipid lowering medication may reduce the risk for IHD. In primary care, it is essential with risk factor assessment to identify high-risk individuals, and subsequent primary prevention with encouragement of physical activity and a healthy diet, low in saturated fat and with a focus on wholegrain products, vegetables, fruit and fish [11, 32]. A recent study of national trends of different cholesterol measures showed that non-HDL-C and TC/HDL-C have decreased in most Western countries, Japan and South Korea between 1980 and 2015, in contrast to China, where TC/HDL-C and non-HDL-C have increased [33]. Dietary changes in Western countries, and to a certain degree the use of statin medication, are important explaining factors behind this trend. Low levels of the healthy HDL-C are associated with obesity and intake of trans-fats and carbohydrates, whereas high levels are associated with physical activity, alcohol consumption and intake of total and unsaturated fat [33–37]. Several randomised controlled trials have concluded that changing dietary saturated fat to polyunsaturated vegetable oil is associated with an improved lipoprotein profile and may reduce the risk of cardiovascular disease by 30% [36].

### Strengths and limitations

The population-based sample of women followed during a long follow-up time is a strength of the present study, and so is the comparison between TC/HDL-C and non-HDL-C. The analyses were adjusted for several potential confounding factors assessed at the baseline examination. There are also some limitations of the study. Firstly, the non-response rate of 36% may result in selection bias where the non-responders may have an increased morbidity and mortality rate [38]. Moreover, the study was carried out in a cohort of middle-aged, mainly white women in southern Sweden and hence the results may not be applicable to other ethnicities or geographic areas. Secondly, some of the variables were based on self-reported data, which is usually not as accurate as the clinical assessment variables. Thirdly, although we included several potential confounding factors, it is possible that residual confounding factors may affect the association. Although the women could report use of a limited number of medications, we had no specific information about lipid lowering medication; however, during the 1990s statins were not recommended in Sweden as primary prevention of cardiovascular disease. Finally, a problem with long-term follow-up studies is that the habits may change over time. However, it has previously been shown that most adults with elevated blood lipids early in life continue to have high levels over their lifecourse [39].

### Conclusions

TC/HDL-C ratio and non-HDL-C are both predictors for IHD in middle-aged women, with a slightly higher predictive ability for TC/HDL-C ratio. However, non-HDL-C was linearly related to IHD and may be easier to calculate and interpret in clinical practice. The use of TC/HDL-C ratio or non-HDL-C is an important part of risk assessment for early prediction of IHD in middle-aged women.

## Data Availability

The data that support the findings of this study are available from the Swedish National Board of Health and Welfare and the Center for Primary Health Care Research, but restrictions apply to the availability of these data, which were used under license for the current study, and so are not publicly available. Data are however available from the authors upon reasonable request and with permission of the Swedish National Board of Health and Welfare and the Center for Primary Health Care Research.

## Abbreviations

IHD: Ischemic heart disease
CI: Confidence interval
HDL-C: High-density lipoprotein cholesterol
HR: Hazard ratio
LDL-C: Low-density lipoprotein cholesterol
TC: Total cholesterol
TC/HDL-C: Total cholesterol to HDL-C ratio
Non-HDL-C: Total cholesterol minus HDL-C
WHILA: Women’s Health in Lund Area
WHR: Waist hip ratio
